# Overcoming the blood-brain barrier: targeted delivery strategies for gliomas

**DOI:** 10.3389/fphar.2025.1705234

**Published:** 2025-11-24

**Authors:** Xue Du, Chunbao Chen, Yijun Zeng, Zhinei Lin

**Affiliations:** 1 Department of Oncology and Hematology, The 3RD Affiliated Hospital of Chengdu Medical College, Chengdu Pidu District People’s Hospital, Chengdu, China; 2 Department of Neurosurgery, The 3RD Affiliated Hospital of Chengdu Medical College, Chengdu Pidu District People’s Hospital, Chengdu, China

**Keywords:** neurological glioma, drug delivery systems, blood-brain barrier, blood-tumor barrier, nanocarriers, targeted therapy

## Abstract

Neurological gliomas, as the most common and deadly primary brain tumors, face two major therapeutic obstacles: the blockade of the blood-brain barrier (BBB) and high tumor heterogeneity. In recent years, research on novel drug delivery systems has brought hope for glioma treatment. This article elaborates on the research progress of novel drug delivery systems in glioma treatment, including various nanocarriers, targeted delivery strategies, and gene therapy drug delivery systems. It analyzes their advantages and challenges, outlooks future development directions, and aims to provide a reference for optimizing drug delivery systems for glioma therapy.

## 1 Introduction

Neurological gliomas originate from glial cells and are the most common primary malignant tumors in the adult central nervous system (CNS). According to the 2021 World Health Organization (WHO) Classification of Tumors of the Central Nervous System, diffuse gliomas are classified into isocitrate dehydrogenase (IDH)-mutant astrocytomas (WHO Grades 2–4), IDH-mutant and 1p/19q codeleted oligodendrogliomas (WHO Grades 2 and 3), and IDH-wildtype glioblastomas (GBM, WHO Grade 4) (Louis et al., 2021). The clinical treatment regimen for brain gliomas involves comprehensive therapy including surgical resection, radiotherapy, and chemotherapy (Stupp et al., 2005). Due to the infiltrative growth and unclear boundaries of gliomas, complete resection is difficult to achieve. Simultaneously, the existence of the Blood-Brain Barrier (BBB) and the Blood-Tumor Barrier (BTB) hinders the entry of anti-tumor drugs into the brain to exert efficacy, leading to slow progress in glioma treatment (van Tellingen et al., 2015; Chen et al., 2023).

The BBB is a highly selective barrier between the blood and brain tissue, restricting the transmembrane transport of many drugs, making it difficult for approximately 98% of small molecule drugs and almost all large molecule drugs to enter the brain parenchyma (Pardridge, 2005). Furthermore, neurological gliomas can induce the formation of a blood-tumor barrier (BTB), further impeding drug delivery (van Tellingen et al., 2015). Therefore, developing efficient drug delivery systems to break through the limitations of the BBB and BTB and achieve effective drug enrichment at the tumor site is key to improving the therapeutic outcome of neurological gliomas.

## 2 Challenges in glioma treatment

### 2.1 Blood-brain barrier and blood-tumor barrier

The existence of the BBB is a major obstacle in glioma treatment. Its endothelial cells have tight junctions, lack fenestrations, possess highly expressed efflux transporters such as P-glycoprotein and breast cancer resistance protein, and contain abundant enzyme systems, all working together to prevent drugs from entering the brain (Obermeier et al., 2013; Kadry et al., 2020). Even if some drugs can cross the BBB, the amount reaching the tumor site is extremely low, often failing to achieve effective therapeutic concentrations. Although the BTB has higher permeability than the BBB in some aspects, it also has its own complexities. The abnormal structure and function of tumor vessels lead to heterogeneous permeability of the BTB, resulting in uneven distribution of drugs within the tumor tissue (Chen et al., 2023). Simultaneously, the BTB retains some of the barrier properties of the BBB, further increasing the difficulty of drug delivery (Arvanitis et al., 2020).

### 2.2 Tumor heterogeneity

Neurological gliomas are highly heterogeneous, exhibiting significant differences in molecular characteristics, biological behavior, and response to therapy among different patients, different regions of the same tumor, and different tumor cell subpopulations (Nicholson and Fine, 2021; Eschbacher et al., 2021). This heterogeneity makes it difficult for a single treatment regimen to be effective against all tumor cells, easily leading to tumor recurrence and drug resistance (Nicholson and Fine, 2021).

### 2.3 Limitations of Traditional therapeutic drugs

Traditional chemotherapeutic drugs like temozolomide, while capable of inhibiting tumor cell growth to some extent, lack targeting specificity. While acting on tumor cells, they also cause toxic side effects on normal tissue cells (Choi et al., 2018). Furthermore, long-term use of chemotherapeutic drugs can easily induce drug resistance in tumor cells, reducing treatment efficacy. Some novel therapeutic drugs, such as gene therapy drugs (Umemura et al., 2023) and immunotherapy drugs (Xu et al., 2020), although potentially highly effective and specific, face drug delivery challenges and struggle to reach tumor cells effectively to exert their effects (Song et al., 2024).

## 3 Research progress on novel drug delivery systems

### 3.1 Nanocarriers

#### 3.1.1 Liposomes

Liposomes are vesicles composed of phospholipids and other lipid materials with bilayer or multilayer membrane structures. They possess good biocompatibility, biodegradability, and low immunogenicity (Large et al., 2021). Liposomes can encapsulate both hydrophilic and hydrophobic drugs, increasing drug solubility and protecting drugs from degradation by enzymes and other components in body fluids (Almeida et al., 2020). Modifying the liposome surface, such as by attaching polyethylene glycol (PEG) to form PEGylated liposomes, can prolong their circulation time in the blood and reduce phagocytosis by the mononuclear phagocyte system (Tenchov et al., 2023). Further modification with targeting ligands, such as mannose (MAN) (Li et al., 2019) or transferrin (Tf) (Dos Santos Rodrigues et al., 2019), enables targeted recognition of specific receptors on tumor cells or the BBB (Eroğlu and İbrahim, 2020).

In delivering nucleic acid drugs, cationic liposomes can bind negatively charged siRNA through electrostatic interactions, achieving siRNA loading (Hu et al., 2024). However, cationic liposomes suffer from cytotoxicity and poor *in vivo* stability. The addition of PEG chains can improve their pharmacokinetic properties but may also limit the uptake of liposomes by tumor cells (Yin et al., 2022). Meng JL et al. developed a pH-responsive anti-polyethylene glycol (PEG) × anti-TfR bispecific antibody (pH-PEG engager ^TfR^). This antibody can complex with PEGylated nanomedicine at physiological pH to trigger TfR-mediated transcytosis in brain microvascular endothelial cells, while rapidly dissociating from the PEGylated nanomedicine at acidic endosomes for efficient release of the nanomedicine to cross the BBB (Meng et al., 2025). The conditional release of PEGylated nanomedicine during BBB-related receptor-mediated transcytosis by pH-pH-PEG engager ^TfR^ is promising for enhanced brain drug delivery to treat CNS disorders. Although such smart delivery systems have demonstrated promising performance in preclinical studies, significant variations in the relative efficacy of different liposomal systems persist. The feasibility of large-scale production and batch-to-batch consistency remain major obstacles to clinical translation.

#### 3.1.2 Polymeric nanoparticles

Polymeric nanoparticles are nanoscale particles prepared from synthetic or natural polymers (Sofini et al., 2024). Commonly used synthetic polymers include polylactic acid (PLA) (Tyler et al., 2016), poly (γ-glutamic acid) (PGA) (Zhang et al., 2024), and their copolymer poly (lactic-co-glycolic acid) (PLGA) (Danhier et al., 2012). Natural polymers include chitosan (Kluczka, 2023) and gelatin (Santoro et al., 2014). Polymeric nanoparticles have good stability, tunable size, and surface properties, enabling effective drug encapsulation and controlled release (Gu et al., 2024).

PLGA nanoparticles exhibit good biocompatibility and biodegradability. Surface modification with different targeting ligands can achieve targeted delivery to gliomas (Das et al., 2023). Chitosan is a natural cationic polysaccharide with good biocompatibility, bioadhesion, and degradability (Abourehab et al., 2022). Chitosan nanoparticles can bind negatively charged drugs or biomacromolecules through electrostatic interactions for loading and delivery (Path et al., 2023). Lakkadwala S *et al.* (Lakkadwala and Singh, 2019) demonstrated that liposomes surface-modified with transferrin (Tf) for receptor targeting and with cell-penetrating peptide PFVYLI (PFV) increased the translocation of doxorubicin (Dox) and erlotinib (Erlo) across the BBB into glioblastoma (U87) tumor cells. Using an *in vitro* brain tumor model, the translocation of dual-functionalized liposomes across the BBB was significantly (*p* < 0.05) higher, delivering chemotherapeutic drugs to the glioblastoma tumor cells inside a PLGA-Chitosan scaffold and resulting in approximately 52% tumor cell death. Although these results are encouraging, multiple challenges arise in actual clinical translation. First, the preparation process for the dual-modified system is complex, making it difficult to ensure batch-to-batch consistency. Second, *in vitro* models cannot fully replicate the intricate *in vivo* BBB microenvironment. Finally, non-specific uptake of the cell-penetrating peptide may induce toxic reactions in normal brain tissue. These issues partially explain why such complex systems struggle to advance into clinical research phases.

#### 3.1.3 Protein nanocarriers

Protein nanocarriers possess good biocompatibility, low immunogenicity, and precise structure (Elzoghby et al., 2012). Human heavy-chain ferritin (HFn) is a well-studied protein nanocarrier. HFn has properties allowing specific tumor recognition and BBB crossing (Kim et al., 2024). To address the issue of lysosomal escape when ferritin delivers siRNA, researchers developed a class of internally cationic HFn variants (HFn + NPs). The top-performing candidate, HFn2, effectively delivered siRNA to glioma cells after traversing the BBB and achieved the highest silencing efficacy among HFn + NPs (Yuan et al., 2022). HFn can selectively deliver a large amount of cargo into tumors *in vivo* via transferrin receptor 1 (TfR1)-mediated tumor-cell-specific targeting followed by rapid internalization. Utilizing the intrinsic tumor-targeting property and unique nanocage structure of human HFn, a broad variety of cargo-loaded HFn formulations have been developed for biological analysis, imaging diagnosis, and medicine development (Song et al., 2021). Although HFn2 demonstrated the highest gene silencing efficacy in preclinical studies, the translation of this protein nanocarrier from laboratory to clinical settings faces unique challenges. These include high costs associated with large-scale production, storage stability issues, and potential immunogenicity risks. Even with human-derived proteins, individual variations may trigger immune responses. Furthermore, the TfR1 expression required for HFn function exhibits heterogeneity across patients and tumor types, potentially limiting its broad applicability.

#### 3.1.4 Inorganic nanoparticles

Inorganic nanoparticles, such as gold nanoparticles, silver nanoparticles, silica nanoparticles, and magnetic nanoparticles, possess unique physicochemical properties and show potential application value in drug delivery (Kumar et al., 2023). Gold nanoparticles have good biocompatibility, high stability, and surface modifiability. They can be used for photothermal therapy via surface plasmon resonance effects and also serve as drug carriers for loading chemotherapeutic drugs or nucleic acid drugs (Zhang et al., 2023). Modifying gold nanoparticles with cell-penetrating peptides can increase their accumulation in the brain for glioma treatment (Griveau et al., 2022).

Magnetic nanoparticles can move directionally and accumulate at the tumor site under an external magnetic field, enabling targeted drug delivery (Gobbo et al., 2015). Simultaneously, magnetic nanoparticles can be used for magnetic resonance imaging (MRI), allowing real-time monitoring of the drug delivery process (Sun et al., 2008). Prepared magnetic nanoparticles surface-modified with targeting ligands can effectively aggregate at the glioma site under magnetic field guidance, improving drug therapeutic efficacy (Tong et al., 2025; Bartusik-Aebisher et al., 2025). However, the clinical translation of inorganic nanoparticles faces challenges due to the lack of long-term *in vivo* safety data, potential bioaccumulation risks, and quality control issues in large-scale production. While magnetic nanoparticles surface-modified with targeting ligands can guide targeting to gliomas under magnetic fields, the penetration depth of external magnetic fields in human tissues is limited, significantly reducing targeting efficiency for deep-seated brain tumors. Furthermore, inorganic nanoparticles typically exhibit poor degradability, potentially leading to long-term toxicity concerns.

#### 3.1.5 Systematic challenges in the clinical translation of nanocarriers

Across all types of nanocarriers, the conversion rate from preclinical research to clinical application remains extremely low. This phenomenon stems from multiple factors. First, differences between animal models and human physiology cause many nanocarriers effective in animal studies to perform poorly in humans. Second, large-scale production of nanocarriers faces quality control challenges, as complex surface modification processes struggle to maintain batch consistency in industrial manufacturing. Finally, regulatory bodies impose stringent requirements on the safety and quality of nanomedicines, yet many nanocarrier systems lack sufficient safety data and clear studies on their *in vivo* metabolic pathways.

The coexisting reproducibility issues cannot be overlooked. Many studies have failed to adequately characterize the physicochemical parameters of nanocarriers, such as particle size distribution, surface charge, and drug loading capacity, making it difficult to compare results across different laboratories. The lack of standardized procedures for complex preparation processes and modification strategies, coupled with discrepancies between *in vitro* experimental conditions and *in vivo* environments, collectively contribute to the reproducibility crisis in nanocarrier research.

Future research should place greater emphasis on addressing these translational barriers. By simplifying carrier design, establishing standardized characterization methods, enhancing safety assessments, and conducting more reliable preclinical model studies, we can further advance the progress of nanocarriers toward clinical application.

### 3.2 Targeted delivery strategies

#### 3.2.1 Passive targeting

Passive targeting primarily utilizes the enhanced permeability and retention (EPR) effect of tumor tissue (Torchilin, 2000). The gaps between tumor vascular endothelial cells are larger, allowing nanocarriers to enter the tumor tissue through these gaps. Concurrently, impaired lymphatic drainage in the tumor region allows nanocarriers to remain in the tumor tissue for an extended period, achieving passive drug accumulation at the tumor site (Sebak et al., 2021). Factors such as the size, shape, surface charge, and hydrophobicity of nanocarriers influence their EPR effect (Zhao et al., 2019). Generally, nanocarriers in the size range of 10–200 nm can better utilize the EPR effect for tumor targeting (Xu et al., 2023). Some drug delivery systems based on liposomes and polymeric nanoparticles have successfully achieved passive targeted delivery to glioma through rational design of nanocarrier size and surface properties (Caro et al., 2021).

However, due to its uneven intensity across various tumor microenvironments, the existence and significance of the EPR effect in nanomedicine design and application have been questioned. The EPR effect exhibits inter-tumor heterogeneity, varying among different patients and different tumor sites. Nanocarriers may also accumulate to some extent in non-tumor tissues, leading to potential toxic side effects (Maeda, 2015). The real challenge now lies in leveraging the EPR effect to design and enhance the therapeutic efficacy of nanomedicine. When considering the EPR effect, maintaining efficacy is crucial, as drugs must sustain effective concentrations within tumor tissues for a sufficient duration to achieve satisfactory anticancer activity.

#### 3.2.2 Active targeting

Active targeting involves modifying the nanocarrier surface with specific targeting ligands, enabling them to bind to specific receptors on tumor cells or the BBB for precise drug delivery (Dutta et al., 2021; Anthony et al., 2021). Commonly used targeting ligands include peptides, antibodies, and aptamers. Peptide ligands have advantages such as small molecular weight, low immunogenicity, and ease of synthesis (Jiang et al., 2019). After modification on the nanocarrier surface, peptides can specifically bind to corresponding receptors, significantly increasing the enrichment of nanocarriers in the brain and at the tumor site (Gao et al., 2014). Antibodies have high specificity and affinity, but their large molecular weight may affect the pharmacokinetic properties of nanocarriers, and they pose immunogenicity issues (Arslan et al., 2021). However, preparing antibody fragments or humanized antibodies can mitigate these problems to some extent (Hillman et al., 2019). Aptamers are single-stranded nucleic acid molecules obtained through *in vitro* selection techniques that can specifically bind to targets. They offer high affinity, high specificity, and ease of synthesis and modification, showing potential application prospects in active targeted delivery for glioma (Esposito et al., 2018).

### 3.3 Gene therapy drug delivery systems

Gene therapy provides new strategies for glioma treatment, such as delivering small interfering RNA (siRNA) (Jin et al., 2025), short hairpin RNA (shRNA) (Lu et al., 2020), or plasmid DNA (Yao et al., 2015) to silence or regulate tumor-associated genes. However, gene therapy drugs are mostly large biomolecules, carry a negative charge, are susceptible to degradation by nucleases, and find it difficult to cross cell membranes and the BBB (Körbelin et al., 2024). Therefore, its therapeutic efficacy depends on efficient and safe delivery systems. In fact, the various nanocarriers discussed earlier, through targeted design, serve as the core tools for addressing the challenges of gene drug delivery.

Systems such as liposomes, polymeric nanoparticles, and protein nanocarriers are widely used for encapsulating and protecting gene drugs due to their inherent properties or functional modifications (Hassan et al., 2025). Cationic liposomes efficiently condense negatively charged siRNA or DNA through electrostatic interactions, forming lipid nanocomplexes that shield nucleic acids from nuclease degradation (Sun and Lu, 2023). Cationic polymers (e.g., poly (ethyleneimine), chitosan) and engineered cationic protein nanocages (e.g., HFn^+^ NPs) similarly load gene drugs via electrostatic interactions (Liu et al., 2025). Therefore, developing effective delivery systems for gene therapy drugs is crucial. Viral vectors are also commonly used gene delivery tools, such as adenoviruses (de la Nava et al., 2024), adeno-associated viruses (AAV) (Merkel et al., 2017), and lentiviruses (Nivajärvi et al., 2020). However, viral vectors pose potential risks of immune responses, insertional mutagenesis, and difficulties in preparation and purification. While non-viral vectors are safer, their transfection efficiency needs further improvement (Butt et al., 2022). In contrast, non-viral nanocarrier systems offer advantages such as high safety, large payload capacity, and ease of large-scale production. Although their transfection efficiency has historically been lower than that of viral vectors, their delivery efficiency is rapidly improving through the synergistic design of integrated targeting ligands and endosomal escape mechanisms.

### 3.4 Osmotic blood-brain barrier opening

Beyond the targeted modifications of the nanocarriers themselves, physical “door-opening” strategies can also enable trans-barrier delivery of chemotherapeutic drugs or nanoparticles. Among these, osmotic blood-brain barrier opening (OBBBO) induced by intra-arterial infusion of hypertonic solutions facilitates drug delivery to the brain, providing a method for achieving global delivery of therapeutic agents to brain tumors and their surrounding regions (Sato et al., 1998). Osmotic opening of the blood-brain barrier (BBB) most likely is mediated by modification of interendothelial tight junctions, subsequent to shrinkage of cerebrovascular endothelial cells, and not by stimulation of transendothelial vesicular transport or by channel formation (Rapoport and Robinson, 1986). Selective and super-selective intra-arterial cerebral infusion (SIACI/SSIACI) delivers chemotherapy directly to tumor-supplying arteries. Combined with the opening of the permeable blood-brain barrier, this approach significantly increases local tumor drug concentrations (Ferreira et al., 2025). A recent study proposed a novel method for opening the BBB by combining 25% mannitol with 4% sodium chloride, significantly enhancing BBB permeability while demonstrating favorable safety profiles in mouse experiments (Qiao et al., 2025).

Although intra-arterial hypertonic mannitol infusion has established itself as a clinically viable technique for opening the blood-brain barrier, its non-selective opening mechanism may allow neurotoxic substances to enter the brain alongside therapeutic agents. Furthermore, the brief duration of the opening window limits its efficacy and scope of application (Bellavanc et al., 2008). However, by integrating this mature permeable open technology with meticulously designed nanomedicine delivery systems and advanced imaging-guided techniques, it establishes a complete “open-delivery-monitoring” technological closed-loop system, demonstrating tremendous synergistic potential and innovative prospects.

### 3.5 Emerging physical/mechanical barrier opening strategy

Compared to biochemical approaches, emerging physical and mechanical strategies directly apply mechanical forces generated by external physical fields to the blood-brain barrier, achieving its opening. These methods offer unique advantages including high spatio-temporal precision, reversible opening effects, and generally not relying on specific cell receptors or pathways, thereby pioneering novel pathways for central nervous system drug delivery.

#### 3.5.1 Focused ultrasound technology

Microbubble-mediated focused ultrasound (Mb-FUS) is a promising non-invasive technique for BBBO, enhancing drug delivery and immunomodulation for brain disease treatments (Bae et al., 2025). The principle involves intravenous injection of micron-sized ultrasound microbubbles, followed by precise excitation of microbubble oscillations in specific brain regions using focused ultrasound. This mechanical oscillation induces multiple biological effects on vascular endothelial cells, including mechanically stretching tight junctions through stable cavitation effects and activating relevant signaling pathways, thereby reversibly increasing blood-brain barrier permeability (Ibsen et al., 2013; McMahon et al., 2019). Combined with transcranial MR-guided focused ultrasound (MRgFUS), this technology enables millimeter-level precision in opening the blood-brain barrier (Meng et al., 2021) and has been widely applied in clinical research to enhance the intracerebral delivery of therapeutic antibodies for Alzheimer’s disease (Bae et al., 2024) and Parkinson’s disease (Meng et al., 2022). Its safety and feasibility have been validated through multiple human trials. Numerous preclinical studies have validated the efficacy of FUS-MB in animal GBM models (Sun et al., 2017; Englander et al., 2021). Meanwhile, several clinical trials have established the safety and feasibility of FUS therapy for glioblastoma, and have demonstrated that FUS can induce immunomodulatory effects (Chen et al., 2021). Furthermore, repeated FUS-mediated BBBO concurrently with radiotherapy is safe and feasible. FUS combined with radiotherapy may improve glioma treatment outcomes, but further studies are needed to optimize the magnitude of effect (Tazhibi et al., 2024; Fletcher et al., 2024).

#### 3.5.2 Convection-enhanced drug delivery

Convection-enhanced delivery (CED) is an invasive local drug administration technique. It employs a slowly and continuously applied fluid infusion pressure within tumor cavities or brain parenchyma via intracranially implanted catheters, thereby generating a “convective” field that drives therapeutic drugs to distribute extensively and uniformly within tissue interstices (Mehta et al., 2017). CED technology can bypass the blood-brain barrier to deliver high-concentration drugs directly to target areas, significantly increasing the volume of distribution. It is particularly suitable for the localized treatment of macromolecular drugs and has undergone extensive clinical exploration in the treatment of diseases such as malignant glioma (Nordling-David et al., 2017; Zhan and Wang, 2018). Early trials lacked effective methods for real-time monitoring of drug distribution within the brain, making it impossible to promptly adjust infusion parameters to ensure complete coverage of the target area. Additionally, there was a lack of long-term efficacy assessment (D'Amico et al., 2021). With the maturation of real-time image guidance key technologies, currently conducted dose-escalation clinical trials utilize MR-guided stereotactic (CED) techniques (Souweidane et al., 2025; Narsinh et al., 2025). Real-time MRI enables surgeons to dynamically adjust infusion parameters based on the actual distribution of the drug within the patient’s brain, achieving truly personalized drug delivery. This ensures that the tumor region in each patient is fully saturated with the medication.

#### 3.5.3 Laser interstitial thermal therapy

Laser interstitial thermal therapy (LITT) is a minimally invasive surgical technique commonly used for the ablation of brain tumors (Carpentier et al., 2012). At energy parameters below those causing irreversible tissue coagulation necrosis, the sublethal thermal energy generated by LITT can temporarily disrupt the blood-brain barrier structure in the irradiated region (Mo et al., 2021). This thermal effect may achieve opening of the blood-brain barrier by influencing tight junction proteins or the cytoskeleton, creating synergistic opportunities for subsequent drug delivery within the hyperthermia zone (Sabel et al., 2003).

#### 3.5.4 Photodynamic therapy

Photodynamic therapy (PDT) utilizes photosensitizers and laser irradiation at specific wavelengths to generate reactive oxygen species, thereby killing tumor cells. Research has confirmed that under specific low-dose photodynamic therapy conditions, the reactive oxygen species produced can trigger inflammatory responses in vascular endothelial cells and the reorganization of tight junctions, leading to a temporary opening of the blood-brain barrier without causing widespread cell death (Semyachk et al., 2018; Semyachkina-Glushkovskaya et al., 2023). This window of opportunity provides a valuable chance to enhance the brain accumulation of subsequent chemotherapy drugs, targeted therapies, and even nanomedicine delivery systems (Li et al., 2025). Synergistic strategy of PDT and nanodelivery systems, this approach employs PDT as the initial intervention. Following intravenous injection of photosensitizers and localized light irradiation to induce BBB opening, mainstream chemotherapy drugs or targeted nanomedicines are subsequently administered (Guo et al., 2025; Chen et al., 2024). Sequential-controlled combination therapy—this “open the door first, then deliver the cargo” approach—significantly enhances the accumulation of subsequent drugs at tumor sites, achieving synergistic therapeutic effects where 1 + 1>2. Of course, future work will also need to optimize light exposure parameters and photosensitizer dosage to strike the optimal balance between opening the blood-brain barrier and causing permanent vascular damage, as well as address the limited penetration of light into deep brain tissues.

## 4 Challenges and prospects

### 4.1 Challenges

Although novel drug delivery systems have made progress in glioma treatment research, they still face many challenges. Firstly, the large-scale preparation and quality control of nanocarriers are difficult; achieving efficient, low-cost, and reproducible nanocarrier production is an urgent problem to be solved (Dahiya et al., 2021; Alshawwa et al., 2022). Secondly, the *in vivo* safety and long-term biological effects of nanocarriers are not yet fully understood; nanomaterials may aggregate or undergo abnormal metabolism in the body, posing potential hazards (Xuan et al., 2020). Furthermore, although various targeting strategies have been proposed, achieving completely precise targeted delivery in practical applications remains challenging; drug distribution in non-target tissues may cause toxic side effects (Ezike et al., 2023). Moreover, due to the high heterogeneity of gliomas, a single drug delivery system and treatment strategy may not meet the needs of all patients (Nicholson and Fine, 2021; Song et al., 2024).

### 4.2 Future prospects

Future research on novel drug delivery systems for glioma treatment could focus on the following aspects. First, further optimize the design of nanocarriers, developing smarter and more efficient ones, such as responsive nanocarriers that can achieve precise drug release and targeted delivery based on changes in the tumor microenvironment (Zhao et al., 2021). Second, deeply research the interaction mechanisms between nanocarriers and biological systems, clarifying their *in vivo* metabolic pathways and long-term safety to provide a solid theoretical foundation for clinical application (Deb and Jain, 2024). Third, combine multimodal treatment strategies, integrating drug delivery systems with immunotherapy, photothermal therapy, and radiotherapy to exert synergistic therapeutic effects and improve glioma treatment outcomes (Huang et al., 2021; Molinaro et al., 2024). Fourth, utilize artificial intelligence and big data technologies to achieve personalized treatment for glioma patients, tailoring suitable drug delivery plans and treatment strategies based on the molecular characteristics of the patient’s tumor and individual differences (Noury et al., 2025). Through continuous research and innovation, more effective drug delivery systems for glioma treatment are expected to be developed, improving patient prognosis.

## 5 Conclusion

The drug delivery challenges faced in glioma treatment severely constrain the improvement of therapeutic efficacy. Novel drug delivery systems, particularly research on various nanocarriers based on nanotechnology and targeted delivery strategies, provide new avenues to address this challenge. Nanocarriers such as liposomes, polymeric nanoparticles, protein nanocarriers, and inorganic nanoparticles demonstrate unique advantages in improving drug solubility and stability, breaking through the blood-brain barrier, and achieving tumor-targeted delivery. The application of passive and active targeting strategies further enhances the precision of drug delivery. Research on gene therapy drug delivery systems also brings new hope for glioma treatment. However, this field still faces numerous challenges, requiring in-depth research on nanocarrier preparation, safety evaluation, improvement of targeting precision, and personalized therapy. It is believed that with continuous research advancements, novel drug delivery systems will bring revolutionary breakthroughs in glioma treatment, significantly improving patients’ quality of life and prognosis.
